# Warm White Light-Emitting Diodes Based on a Novel Orange Cationic Iridium(III) Complex

**DOI:** 10.3390/ma10060657

**Published:** 2017-06-16

**Authors:** Huaijun Tang, Guoyun Meng, Zeyu Chen, Kaimin Wang, Qiang Zhou, Zhengliang Wang

**Affiliations:** Key Laboratory of Comprehensive Utilization of Mineral Resources in Ethnic Regions, Joint Research Centre for International Cross-border Ethnic Regions Biomass Clean Utilization in Yunnan, School of Chemistry & Environment, Yunnan Minzu University, Kunming 650500, China; mengguoyun@sina.com (G.M.); chenzy1120@163.com (Z.C.); kmwang041684@163.com (K.W.); xchow123@126.com (Q.Z.)

**Keywords:** cationic iridium(III) complex, warm whitelight-emitting diode, photoluminescence, blue GaN chip, YAG:Ce

## Abstract

A novel orange cationic iridium(III) complex [(TPTA)_2_Ir(dPPOA)]PF_6_ (TPTA: 3,4,5-triphenyl-4*H*-1,2,4-triazole, dPPOA: N,N-diphenyl-4-(5-(pyridin-2-yl)-1,3,4-oxadiazol-2-yl)aniline) was synthesized and used as a phosphor in light-emitting diodes (LEDs). [(TPTA)_2_Ir(dPPOA)]PF_6_ has high thermal stability with a decomposition temperature (*T*_d_) of 375 °C, and its relative emission intensity at 100 °C is 88.8% of that at 25°C. When only [(TPTA)_2_Ir(dPPOA)]PF_6_ was used as a phosphor at 6.0 wt % in silicone and excited by a blue GaN (GaN: gallium nitride) chip (450 nm), an orange LED was obtained. A white LED fabricated by a blue GaN chip (450 nm) and only yellow phosphor Y_3_Al_5_O_12_:Ce^3+^ (YAG:Ce) (1.0 wt % in silicone) emitted cold white light, its CIE (CIE: *Commission International de I’Eclairage*) value was (0.32, 0.33), color rendering index (CRI) was 72.2, correlated color temperature (CCT) was 6877 K, and luminous efficiency (*η_L_*) was 128.5 lm∙W^−1^. Such a cold white LED became a neutral white LED when [(TPTA)_2_Ir(dPPOA)]PF_6_ was added at 0.5 wt %; its corresponding CIE value was (0.35, 0.33), CRI was 78.4, CCT was 4896 K, and *η_L_* was 85.2 lm∙W^−1^. It further became a warm white LED when [(TPTA)_2_Ir(dPPOA)]PF_6_ was added at 1.0 wt %; its corresponding CIE value was (0.39, 0.36), CRI was 80.2, CCT was 3473 K, and *η_L_* was 46.1 lm∙W^−1^. The results show that [(TPTA)_2_Ir(dPPOA)]PF_6_ is a promising phosphor candidate for fabricating warm white LEDs.

## 1. Introduction

Due to high efficiency, long lifetime, and energy-saving and environmentally-friendly properties, white light-emitting diodes (WLEDs) have attracted significant attention and are used as the new generation solid-state light sources in general illumination, full-color displays, liquid crystal display backlights and so on [1,2,3,4]. At present, the commercial WLEDs are mainly obtained by the combination of a blue GaN (GaN: gallium nitride) chip (λ_max,em_ ≈ 450 nm)and a yellow-emitting Y_3_Al_5_O_12_:Ce^3+^ (YAG:Ce) phosphor. YAG:Ce is a blue-light excitable phosphor with high photoluminescence efficiency and good thermal stability [4,5,6]. However, because the main emission of YAG:Ce is in the greenish yellow region, aforementioned commercial WLEDs have low color rendering index (CRI) and high correlated color temperature (CCT). Due to the absence of a red light component in their spectra, the light emitted from such WLEDs is cold white light [7,8,9,10,11,12,13,14,15,16]. Two approaches have been developed to overcome this drawback. In the first approach, a red light component was emitted via some ions (such as Eu^3+^, Pr^3+^) being doped in YAG:Ce [7,8,9]; however, the yellow emission was obviously decreased although slightly red light was obtained. In the other approach, some red phosphors were complementally used in aforementioned WLEDs [5,9,10]. Relatively better performances (including CRI, CCT and efficiency etc.) can be obtained via the second approach, because the red phosphors are mainly excited by the blue GaN chip and the emission of YAG:Ce is seldom affected. Hence, the development of new efficient red phosphors for warm WLEDs based on blue chips is very important and urgently needed. Up to now, besides many inorganic phosphors (such as CaSiAlN_3_:Eu^2+^ [10], InP/GaP/ZnS quantum dots [11], Mn^4+^ activated fluorides [5,12,13], and so on), many organic luminescent materials (such as organic metal complexes [14,15,16,17,18], polymers [19] and small-molecule fluorescent dyes [20,21], and so on) have also been used as red luminescent materials in WLEDs.

Up to now, many types of organic metal complexes (e.g., cationic ruthenium(II) complexes, cationic copper(I) complexes, europium(III) complexes, platinum(II) complexes, zinc(II) complexes, etc.) have been used in various luminescent fields. By comparison, as efficient luminescent materials, cationic iridium(III) complexes have some excellent photochemical and photophysical properties, such as high efficiency of 100% theoretical quantum efficiency, good color tenability via various ligands, short triplet state lifetimes, high thermal and photic stability and so on [22,23]. In the past decade and even earlier, cationic iridium(III) complexes have been widely applied in light-emitting electrochemical cells (LECs) [22,23,24], organic light-emitting diodes (OLEDs) [24,25,26], chemical sensors [27], and bioimaging [28] etc.

Recently, cationic iridium(III) complexes have also been used as luminescence conversion materials (i.e., phosphors) in inorganic LEDs [15,18,29,30], and exhibited good performances. In 2013, C.-Y. Sun et al. [29] achieved efficient white-light emission by encapsulating the cation of a yellow-emitting iridium complex [Ir(ppy)_2_(bpy)]PF_6_ (ppy: 2-phenylpyridine; bpy: 2,2′-bipyridine) at different ratios in theblue-emitting anionic Cd-based metal–organic frameworks (MOF) cavity. When an optimal concentration (3.5 wt %) of [Ir(ppy)^2^(bpy)]^+^ was encapsulated into the host framework and under the excitation of 370 nm ultraviolet light, high-quality white light was obtained with Commission International de I’Eclairage (CIE) coordinates of (0.31, 0.33), a CRI of ca. 80, a CCT of ca. 5900 K, and a quite high quantum yield up to 20.4%. Then high-quality WLEDs were fabricated by using this composite material and ultraviolet chips. In 2016, L. Niklaus et al. [18] fabricated white hybrid light-emitting diodes (WHLEDs) using luminescent rubber-like materials based on a wide palette of compounds including small-molecules, quantum dots, polymers, and coordination complexes. The use of rubbers based on the complex [Ir(ppy)_2_(tb-bpy)]PF_6_ (tb-bpy: 4,4′-ditert-butyl-22,2′-bipyridine) outperformed the others in terms of color quality (CRI > 80) and luminous efficiency (>100 lm·W^−1^) with unprecedented stabilities of more than 1000 h (extrapolated 4000 h) under continuous operation conditions. In one reported work of our research group in 2015 [30], WLEDs fabricated by using cationic iridium(III) complexes [Ir(ppy)_2_(phen)]PF_6_ or [Ir(ppy)_2_(phen)]TiF_6_ (phen: 1,10-phenanthroline) as luminescence conversion materials showed higher CRI and lower CCT than those of the widely used YAG:Ce. In another reported work of our research group also in 2015 [15], a novel red-emitting cationic iridium(III) complex using 2-(9-(2-ethylhexyl)-9H-carbazol-3-yl)benzo[d]thiazole (CBT) as the main ligand and N,N-diphenyl-4-(5-(pyridin-2-yl)-1,3,4-oxadiazol-2-yl)aniline (dPPOA) as the auxiliary ligand was used as a red phosphor in YAG:Ce based WLEDs with 465 nm-emitting GaN blue chips. The WLED only using YAG:Ce as a phosphor at 1.0 wt % emitted cold white; such WLED can become warm WLEDs when the cationic iridium(III) complex was added over 1.0 wt %.

As a consecutive and upgraded research by us, in this work, another novel orange cationic iridium(III) complex also using dPPOA as auxiliary ligand, but using 3,4,5-triphenyl-4H-1,2,4-triazole (TPTA) instead of CBT as the main ligand was synthesized and also used in YAG:Ce-based WLEDs to obtain warm white light. Donor–acceptor bipolar units have been widely used in organic photoresponse materials for extending absorption range and increasing absorption rate [25,31,32]. Many organic cationic iridium(III) complexes can easily be excited by ultraviolet light, but cannot be effectively excited by blue light (such as the blue light of a GaN chip). In order to make this new complex effectively excited by the blue light of a GaN chip, adonor–acceptor bipolar unit of triphenylamine–oxadiazole was contained in the auxiliary ligand dPPOA; theoretically, the electron-donating or/and electron-withdrawing functional groups on the main ligand will further improve the effect of this donor–acceptor bipolar unit [31,32]. In our previous work [15], CBT containing an electron-donating carbazole group had been successfully used in the cationic iridium(III) complex [Ir(CBT)_2_(dPPOA)]PF_6_ for warm WLEDs. This work tried the main ligand TPTA containing an electron-withdrawing 1,2,4-triazole group. In order to achieve higher performance in this new work, besides the replacement of the main ligand in the complex as a new attempt, 450 nm-emitting GaN blue chips with much higher luminous efficiency were also used to replace the 465 nm-emitting GaN blue chips used in previous work [15]. As expected, the cold white light of YAG:Ce-based WLEDs also gradually became warm white light with the increase of the addition of this new cationic iridium(III) complex; at the same time, such WLEDs exhibited encouraging light-emitting performances.

## 2. Materials and Methods

### 2.1. General Information

All chemicals and reagents were purchased from chemical reagent companies and used without further purification unless otherwise stated. ^1^H NMR spectra were recorded on a Bruker AV400 spectrometer (Bruker, Fällanden, Switzerland) operating at 400 MHz; tetramethylsilane (TMS) was used as internal standard. Mass spectra (MS) were obtained on a Bruker amaZon SL liquid chromatography mass spectrometer (LC-MS, Bruker, Karlsruhe, Germany) with an electrospray ionization (ESI) interface using acetonitrile as matrix solvent. Elemental analysis (EA) was performed on a Vario EL III Elemental Analysis Instrument (Elementar, Hanau, Germany). Ultraviolet-visible (UV-vis, Agilent, Palo Alto, CA, USA) absorption spectra were measured on an Agilent 8453 UV-visible Spectroscopy System. Excitation and emission spectra of samples were documented on a Cary Eclipse FL1011M003 (Varian, Palo Alto, CA, USA) spectrofluorometer; the xenon lamp was used as an excitation source, and the temperature of solid samples was controlled by a temperature controller (REX-C110, Kaituo Compressor Parts Co., Ltd, Dongguan, China). Thermogravimetric (TG) analysis was carried out up to 600 °C in N_2_ atmosphere with a heating speed of 10.0 °C·min^−1^ on a NETZSCH STA 449F3 thermogravimetric analyzer (NETZSCH, Selb, Germany). The electroluminescent spectra of LEDs were recorded on a high-accuracy array spectrometer (HSP6000, HongPu Optoelectronics Technology Co., Ltd, Hangzhou, China).

### 2.2. Synthesis

**Figure 1:**
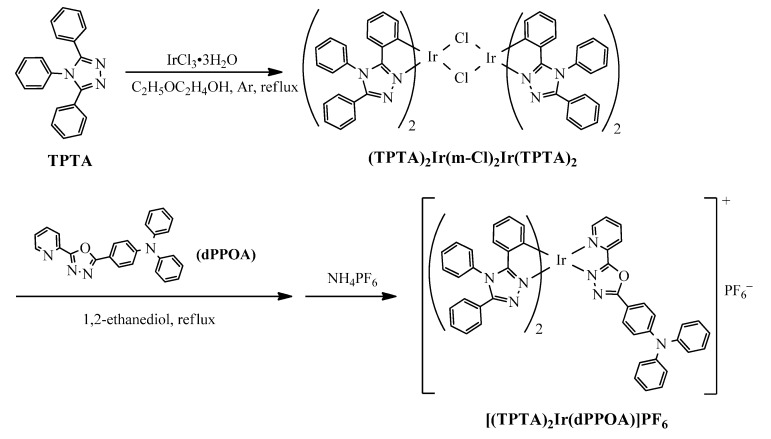
Synthetic route and chemical structure of [(TPTA)_2_Ir(dPPOA)]PF_6_.

Thecationic iridium(III) complex [(TPTA)_2_Ir(dPPOA)]PF_6_ was synthesized by using 3,4,5-triphenyl-4*H*-1,2,4-triazole (TPTA) and N,N-diphenyl-4-(5-(pyridin-2-yl)-1,3,4-oxadiazol-2-yl)aniline (dPPOA) as ligands, as shown in Figure 1. TPTA and dPPOA were synthesized according to the reported procedures in references [33] and [15] respectively.

Synthesis of the chloro-bridged dimer (TPTA)_2_Ir(μ-Cl)_2_Ir(TPTA)_2_: A mixture of IrCl_3_·3H_2_O (1.06 g, 3.0 mmol) and TPTA (1.81 g, 6.10 mmol) in H_2_O (10 mL) and 2-methoxyethanol (30 mL) was refluxed in Ar atmosphere for 24 h. After being cooled to room temperature, the resultant yellow precipitate was collected on a filter, washed with water and methanol alternately, and then dried in a vacuum. Yield was 75.0% (1.85 g) yellow solid. This dimer product was directly used for the next step without further purification and characterization.

Synthesis of the cationic iridium(III) complex[(TPTA)_2_Ir(dPPOA)]PF_6_: The chloro-bridged dimer (TPTA)_2_Ir(μ-Cl)_2_Ir(TPTA)_2_ (0.62 g, 0.38 mmol) and dPPOA (0.30 g, 0.76 mmol) were added into glycol (30 mL) and then kept at 150 °C in Ar atmosphere with stirring for 16 h. After being cooled to room temperature, 10 mL 1.0 mol·L^−1^ aqueous solution of NH_4_PF_6_ was added with stirring, and much orange flocculent precipitate appeared. The precipitate was filtered, washed with water and dried in a vacuum. The crude product was purified by column chromatography on silica gel, eluting with CH_2_Cl_2_/MeCN (volume rate, 10:1). Yield was 86.0% (0.86 g) orange solid. ^1^H NMR (400 MHz, CDCl_3_, 25 °C, ppm, being shown in Figure S1 as electronic supplementary materials), δ: 8.50 (d, 1H, ^3^*J* = 7.6 Hz, ArH), δ: 8.33–8.63 (m, 1H, ArH), δ: 8.16 (d, 1H, ^3^*J* = 5.6 Hz, ArH), δ: 7.97–7.99 (m, 2H, ArH), δ: 7.62–7.69 (m, 7H, ArH), δ: 7.47–7.53 (m, 4H, ArH), δ: 7.31–7.39 (m, 11H, ArH), δ: 7.23–7.27 (m, 3H, ArH), δ: 7.14–7.18 (m, 6H, ArH), δ: 7.04–7.06 (m, 2H, ArH), δ: 6.91–7.00 (m, 2H, ArH), δ: 6.75–6.78 (m, 2H, ArH), δ: 6.71 (t, 1H, ^3^*J* = 7.6 Hz, ArH), δ: 6.66 (d, 1H, ^3^*J* = 7.2 Hz, ArH), δ: 6.44 (t, 2H, ^3^*J* = 7.6 Hz, ArH). MS (*m*/*z*, ESI^+^, being shown in Figure S2 as electronic supplementary materials): calc. for C_65_H_46_F_6_IrN_10_OP, 1320.3, found, 1175.3 [M–PF_6_]^+^. Element analysis calculation for C_65_H_46_F_6_IrN_10_OP: C, 59.13; H, 3.51; N, 10.61%. Found: C, 58.69; H, 3.67; N, 9.91%.

### 2.3. Fabrication and Measurements of LEDs

Two kinds of LEDs were fabricated and measured in this work. (i) Only [(TPTA)_2_Ir(dPPOA)]PF_6_ was blended and stirred homogeneously in silicone atthe mass ratios of 1.0, 2.0, 3.0, 4.0, 5.0 and 6.0 wt %, and then coated on the surface of 450 nm-emitting GaN chips until the reflective cavities were filled up; (ii) YAG:Ce was blended in silicone at a constant mass ratio of 1.0 wt %, and [(TPTA)_2_Ir(dPPOA)]PF_6_ was blended together at different mass ratios of 0.0, 0.5, 1.0 and 1.5 wt %. The silicone containing YAG:Ce and [(TPTA)_2_Ir(dPPOA)]PF_6_ was also coated on the surface of 450 nm-emitting GaN chips until the reflective cavities were filled up. In the blending process, an analytical balance with the readability of 0.01 mg was used for weighing, 0.10000–0.12000 g viscous silicone was weighed in a little bottle every time, then the quantities of [(TPTA)_2_Ir(dPPOA)]PF_6_ and/or YAG:Ce were calculated and weighed. After [(TPTA)_2_Ir(dPPOA)]PF_6_ and/or YAG:Ce were added to the silicone, a drop of CH_2_Cl_2_ was also added, then the mixture was stirred by hand with a little stainless steel rod for about one minute until all the ingredients were well combined. The LEDs were dried and solidified at 150 °C for 1 h. The LEDs were all operated at 20 mA forward current and 5 V reverse voltage, with their performances measured by an integrating sphere spectroradiometer system (Everfine PMS-50, Everfine Photo-E-Info Co., Ltd., Hangzhou, China).

## 3. Results and Discussion

### 3.1. UV-Vis Absorption Spectra

**Figure 2:**
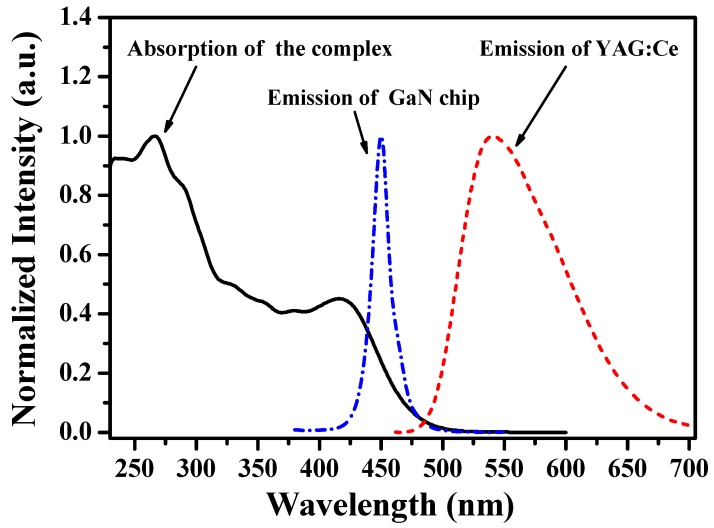
Normalized UV-vis absorption spectrum of [(TPTA)_2_Ir(dPPOA)]PF_6_ in CH_2_Cl_2_ solution at 1.0 × 10^−5^ mol∙L^−1^ and emission spectra of the blue GaN chip and YAG:Ce.

The normalized UV-vis absorption spectrum of [(TPTA)_2_Ir(dPPOA)]PF_6_ in CH_2_Cl_2_ solution at 1.0 × 10^−5^ mol∙L^−1^ together with the emission spectra of the blue GaN chip and YAG:Ce are shown in Figure 2. The strong absorption band between 230 nm and 380 nm can be ascribed to the spin-allowed ^1^π–π* transition of the ligands; the maximum absorption wavelength (λ_abs, max_) is 267 nm (ε_2__67nm_ = 5.4 × 10^4^ L·mol^−1^·cm^−1^). The weak absorption band from 380 nm extending to the visible region results from the overlapping absorption of the spin-allowed singlet metal-to-ligand charge-transfer (^1^MLCT), the spin-forbidden triplet metal-to-ligand charge-transfer (^3^MLCT) and ^3^*π*–π* in the ligands [25,34]. The admixture absorption of ^3^MLCT and ^3^*π*–π* with higher-lying ^1^MLCT is caused by the strong spin-orbit coupling induced by the heavy iridium atom [35,36]. As shown in Figure 2, there is a large overlap between the absorption spectrum of [(TPTA)_2_Ir(dPPOA)]PF_6_ and the emission spectrum of the blue GaN chip (λ_em, max_ = 450 nm), which suggests that Förster resonance energy transfer (FRET) is able to take place between them easily and [(TPTA)_2_Ir(dPPOA)]PF_6_ can be well excited by the blue GaN chip [37]. The overlap between the absorption spectrum of [(TPTA)_2_Ir(dPPOA)]PF_6_ and the emission spectrum of YAG:Ce can almost be ignored, which suggests that the emission of YAG:Ce would be seldom affected by [(TPTA)_2_Ir(dPPOA)]PF_6_, and [(TPTA)_2_Ir(dPPOA)]PF_6_ is a practicable red luminescent additive for YAG:Ce-based WLEDs.

### 3.2. Photoluminescent Properties

**Figure 3:**
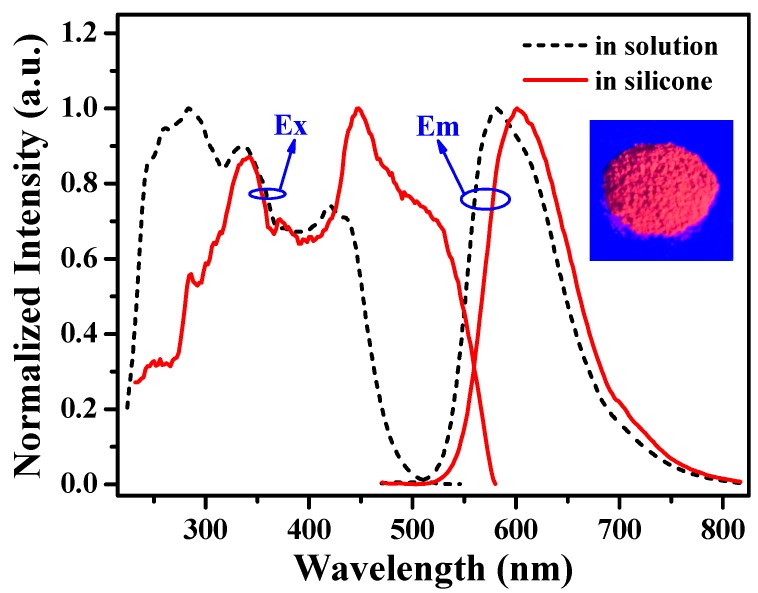
Normalized excitation (Ex, λ_em_ = 581, 600 nm) and emission (Em, λ_ex_ = 450 nm) spectra of [(TPTA)_2_Ir(dPPOA)]PF_6_ in CH_2_Cl_2_ solution at 1.0 × 10^−5^ mol∙L^−1^ and blended in silicone at 6.0 wt % (coated on quartz plate). Inset: A photograph of [(TPTA)_2_Ir(dPPOA)]PF_6_ powders excited by blue light (λ_ex_ = 450 nm).

The photoluminescent properties of [(TPTA)_2_Ir(dPPOA)]PF_6_ in solution and blended in silicone were investigated and the results are shown in Figure 3. Both excitation (Ex, λ_em_ =600 nm) and emission (Em, λ_ex_ = 450 nm) spectra of [(TPTA)_2_Ir(dPPOA)]PF_6_ in silicone showed obvious red shift in comparison with those of [(TPTA)_2_Ir(dPPOA)]PF_6_ in CH_2_Cl_2_ solution (λ_em_ = 581 nm, λ_ex_ = 450 nm). The red shift should be caused mainly by the increase of the conjugated system of [(TPTA)_2_Ir(dPPOA)]PF_6_ in solid silicone, because the steric hindrance and distortion of the phenyls in ligands TPTA and dPPOA are restrained to a certain degree. At the same time, the conjugated system of [(TPTA)_2_Ir(dPPOA)]PF_6_ can also be increased by intermolecular *π–π* stacking in silicone.

In general, sufficient overlap between the excitation spectra of phosphors and the emission spectra of the LED chips is necessary to realize energy transfer from the LED chips to phosphors efficiently. In this work, the excitation spectra of [(TPTA)_2_Ir(dPPOA)]PF_6_ in CH_2_Cl_2_ solution and blended in silicone lie 225−500 nm and 225−580 nm respectively, and both cover the emission spectra of the 450 nm-emitting blue GaN chip (as shown in Figure 2), which means that [(TPTA)_2_Ir(dPPOA)]PF_6_ can be efficiently excited by the blue GaN chip. In particular, there is a maximum wavelength (448 nm) near 450 nm in the excitation spectra of [(TPTA)_2_Ir(dPPOA)]PF_6_ blended in silicone, which suggests that the [(TPTA)_2_Ir(dPPOA)]PF_6_ can be efficiently excited by the blue light of the GaN chip. The emission spectra of [(TPTA)_2_Ir(dPPOA)]PF_6_ mainly lie from 520 nm to 750 nm with the maximum emission wavelengths of 581 nm (in solution) and 600 nm (in silicone) respectively; obviously, considerable red light component was contained in its emission spectra and [(TPTA)_2_Ir(dPPOA)]PF_6_ can be used as a phosphor for fabricating warm WLEDs.

### 3.3. Thermal Stability and Thermal Quenching Properties

**Figure 4:**
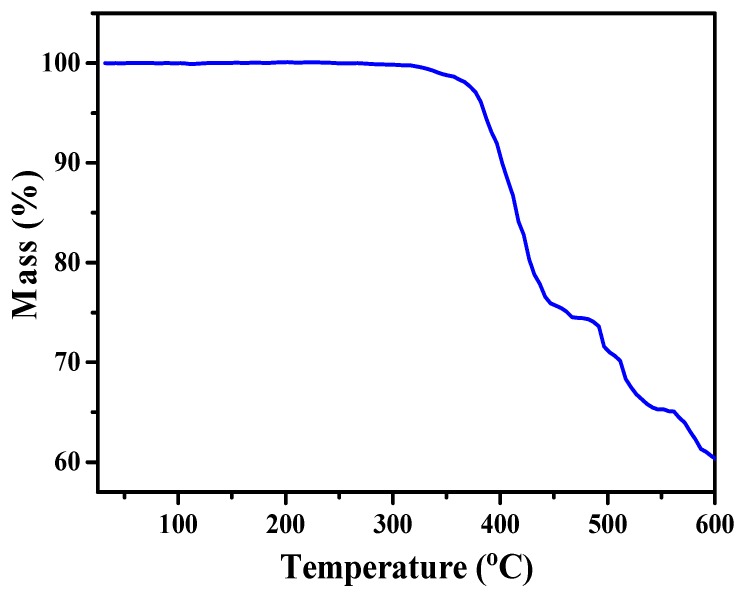
Thermogravimetric (TG)curve of [(TPTA)_2_Ir(dPPOA)]PF_6_.

The thermal property of [(TPTA)_2_Ir(dPPOA)]PF_6_ was characterized by thermogravimetry (TG) in nitrogen atmosphere at a heating speed of 10.0 °C·min^−1^, and the resultant TG curve is shown in Figure 4. With temperature increasing, the TG curve begins to dip suddenly after about 375 °C, which means that the thermal decomposition happened and 375 °C can be regarded asits thermal decomposition temperature (*T*_d_). Such a high decomposition temperature suggests that [(TPTA)_2_Ir(dPPOA)]PF_6_ has a high thermal stability and is enough to meet the requirement of its application in LEDs, since LED devices are fabricated and usually work at a temperature below 150 °C [14].

**Figure 5:**
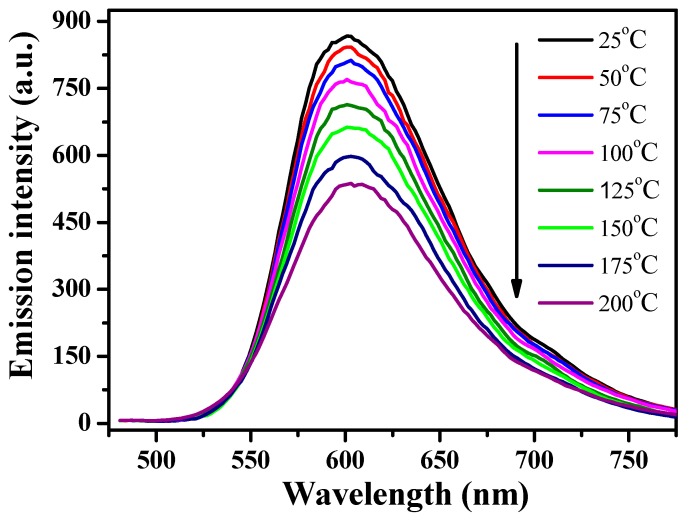
Temperature-dependent photoluminescent emission spectra of [(TPTA)_2_Ir(dPPOA)]PF_6_ powders measured with increasing temperature from 25 °C to 200 °C, λ_ex_ = 450 nm.

**Figure 6:**
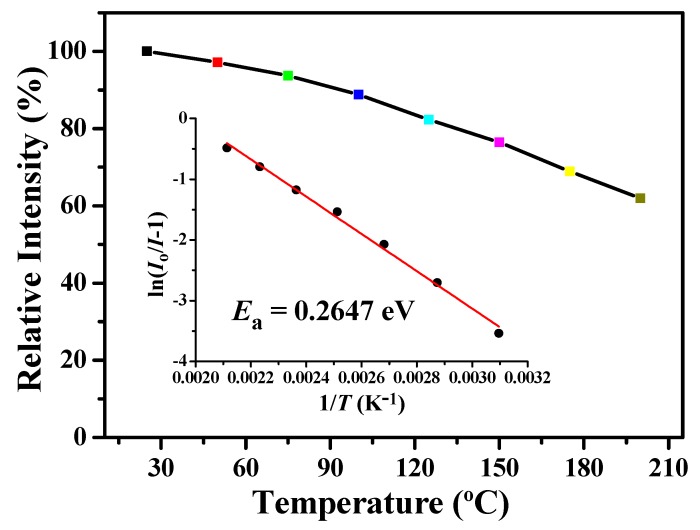
Relative emission intensity of [(TPTA)_2_Ir(dPPOA)]PF_6_ as a function of temperature. The inset represents the ln(*I*_o_/*I* − 1) versus 1/*T* and the calculated activation energy (*E_a_*) for [(TPTA)_2_Ir(dPPOA)]PF_6_.

The thermal-quenching property of [(TPTA)_2_Ir(dPPOA)]PF_6_ was alsoinvestigatedand the results are shown in Figure 5 and Figure 6. Figure 5 depicts the temperature-dependent photoluminescent spectra (λ_ex_ = 450 nm) of [(TPTA)_2_Ir(dPPOA)]PF_6_ powders. From 25 °C to 200 °C, the light-emitting color of [(TPTA)_2_Ir(dPPOA)]PF_6_ exhibited high thermal stability because the profile, wavelength band and the maximum wavelengths (around 601 nm) of its emission spectra at different temperatures were almost unchanged, with the exceptionof the intensity. The relative emission intensity of [(TPTA)_2_Ir(dPPOA)]PF_6_ as a function of temperature is shown in Figure 6. Like most phosphors used in LEDs, the emission intensity of [(TPTA)_2_Ir(dPPOA)]PF_6_ also decreases with increasing temperature (i.e., thermal quenching) [4,38,39,40]. The relative emission intensities at different temperaturesare descending: 97.2% (at 50 °C), 93.7% (at 75 °C), 88.8% (at 100 °C), 82.3% (at 125 °C), 76.5% (at 150 °C), 68.9% (at 175 °C), 61.9% (at 200 °C). The thermal quenching of luminescent organic metal complexes is usually caused by the aggravating jiggle and wiggle of atoms, and the rotating and stretching vibration of covalent bonds as the temperature increases. The activation energy (*E_a_*) of the thermal quenching can be described by the Arrhenius equation [38,39,40]:

$$I=\frac{I_{o}}{1+A\exp(-\frac{E_{a}}{k_{B}T})}$$

where *I* represents the emission intensity at any testing temperature (25–200 °C), *I_o_* represents the emission intensity at room temperature (25 °C), *A* is a constant, *k_B_* is a Boltzmann constant, and *T* is any testing temperature. From the Arrhenius equation, the relationship of ln(*I_o_*/*I* − 1) with 1/*T* can be obtained; the experimental data are well-fitted and shown in Figure 6 (inset); then, the value of *E_a_* for [(TPTA)_2_Ir(dPPOA)]PF_6_ was calculated to be 0.2647 eV from the slope value −(*E_a_*/*k_B_*). For phosphors used in LEDs, a high *E_a_* value means low thermal quenching. The emission intensity decay rate and *E_a_* value show that [(TPTA)_2_Ir(dPPOA)]PF_6_ is an applicable phosphor for LEDs and its thermal quenching is lower than that of many orange or red phosphors reported in recent years [40,41,42].

### 3.4. Fabrication and Performance of LEDs

**Figure 7:**
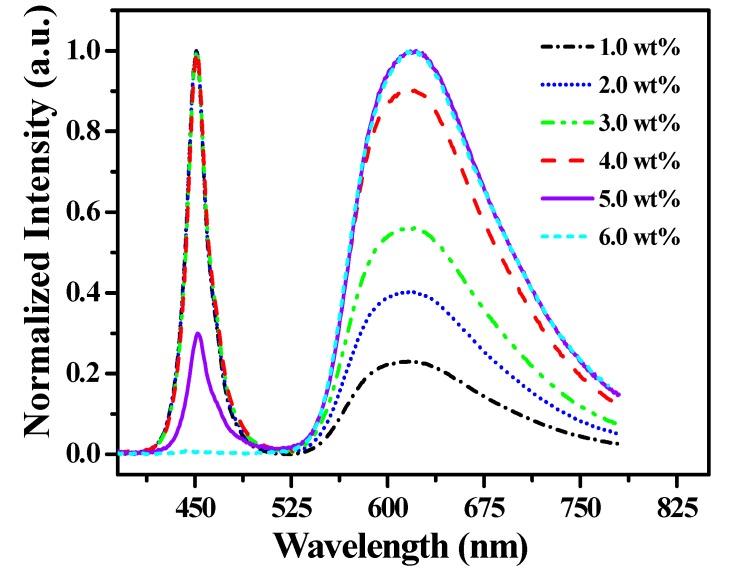
Emission spectra of light-emitting diodes (LEDs) using blue GaN as chips and only [(TPTA)_2_Ir(dPPOA)]PF_6_ as a phosphor at different blending concentrations at 20 mA forward current.

At first, in order to understand the luminescent property of [(TPTA)_2_Ir(dPPOA)]PF_6_ itself in LEDs, a series of LEDs fabricated using blue GaN (450 nm) as chips and only [(TPTA)_2_Ir(dPPOA)]PF_6_ as a phosphor at different blending concentrations were investigated. The emission spectra of such LEDs at 20 mA forward current are shown in Figure 7 and the performances are listed in Table 1. Except for the emission spectrum of the LED using [(TPTA)_2_Ir(dPPOA)]PF_6_ at 6.0 wt %, two emission peaks were contained in the emission spectra of the other LEDs. Obviously, the blue emission peaks on the left with the maximum wavelengths around 450 nm were the emission of the blue GaN chips, which were not completely absorbed by [(TPTA)_2_Ir(dPPOA)]PF_6_ at low blending concentrations. The broad emission peaks (550–770 nm) on the right with the maximum wavelengths around 615 nm can be ascribed to the emission of [(TPTA)_2_Ir(dPPOA)]PF_6_ because they were primarily consistent with the PL spectra of [(TPTA)_2_Ir(dPPOA)]PF_6_. The blending concentrations of [(TPTA)_2_Ir(dPPOA)]PF_6_ increased from 1.0 wt % to 6.0 wt %, while the emission peaks of blue GaN chips gradually declined and eventually disappeared at 6.0 wt %. On the other hand, the emission peaks of [(TPTA)_2_Ir(dPPOA)]PF_6_ gradually increased; at 6.0 wt %, only orange light of [(TPTA)_2_Ir(dPPOA)]PF_6_ emitted from the LED; the CIE (Commission Internationale de L’Eclairage) chromaticity coordinates of this LED was (0.62, 0.38), the CRI was 48.0 and the CCT was 1265 K. These results show that [(TPTA)_2_Ir(dPPOA)]PF_6_ can be efficiently excited by blue GaN (450 nm) chips, and it is a potential red-light-source phosphor for changing the cold white light of aforementioned YAG:Ce-based WLEDs into warm white light.

**Figure 8:**
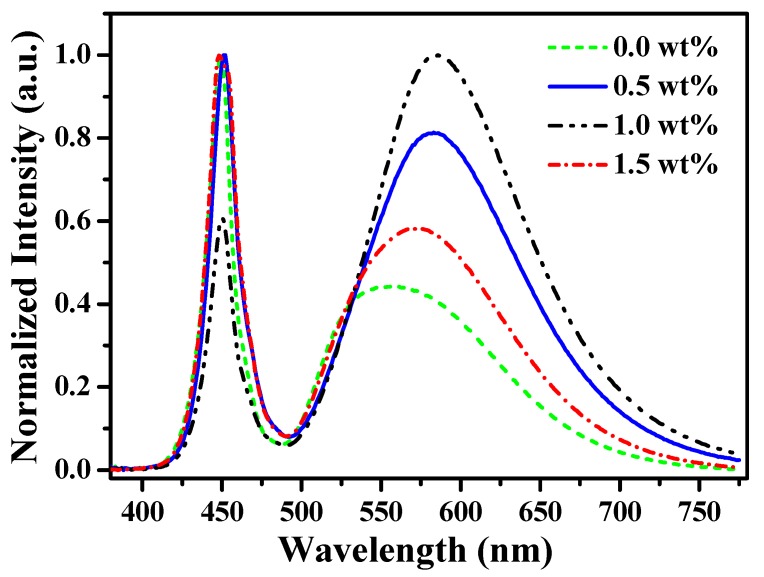
Emission spectra of blue GaN-based white light-emitting diodes (WLEDs) using YAG:Ce (1.0 wt %) and [(TPTA)_2_Ir(dPPOA)]PF_6_ (*x* wt %, *x* = 0.0, 0.5, 1.0, 1.5) as phosphors blended insilicone at different concentrations.

In order to demonstrate the application of [(TPTA)_2_Ir(dPPOA)]PF_6_ for WLEDs, a series of blue GaN-based WLEDs using YAG:Ce (1.0 wt %) and [(TPTA)_2_Ir(dPPOA)]PF_6_ (*x* wt %, *x* = 0.0, 0.5, 1.0, 1.5) as phosphors blended in silicone were fabricated and measured. The emission spectra of these WLEDs are shown in Figure 8 and their performances are listed in Table 2. As mentioned previously, the emission peaks of blue GaN chips were also on the left, and their maximum wavelengths were also around 450 nm. On the right, the broad emission peaks from 500 nm to 725 nm were the mixture of emissions from YAG:Ce and [(TPTA)_2_Ir(dPPOA)]PF_6_ at different concentrations respectively. Before [(TPTA)_2_Ir(dPPOA)]PF_6_ was blended in, the WLED (No. g) only using YAG:Ce as its phosphor exhibited high CCT (6877 K) and low CRI (72.2). After [(TPTA)_2_Ir(dPPOA)]PF_6_ wasblended in, the emission peaks on the right showed visible red shift and became broader. When the blending concentrations increased from 0.0 wt % to 1.5 wt %, the maximum wavelengths changed from 554 nm (No. g) to 571 nm (No. h), and then to 582 nm (No. i) and 586 nm (No. j); at the same time, the intensity of the right emission peaks also increased with the increase of the blending concentrations of [(TPTA)_2_Ir(dPPOA)]PF_6_. The correlated color temperatures (CCTs) of WLEDs using [(TPTA)_2_Ir(dPPOA)]PF_6_ at 0.5 wt % (No. h), 1.0 wt % (No. i) and 1.5 wt % (No. j) were 4896 K, 3473 K and 2864 K respectively; obviously, the CCTs declined in order with the increase of blending concentrations. The CRIs of WLEDs No. h, i and j were 78.4, 80.2 and 75.7 respectively; all of them were higher than the CRI (72.2) of the WLED No. g without [(TPTA)_2_Ir(dPPOA)]PF_6_.

**Figure 9:**
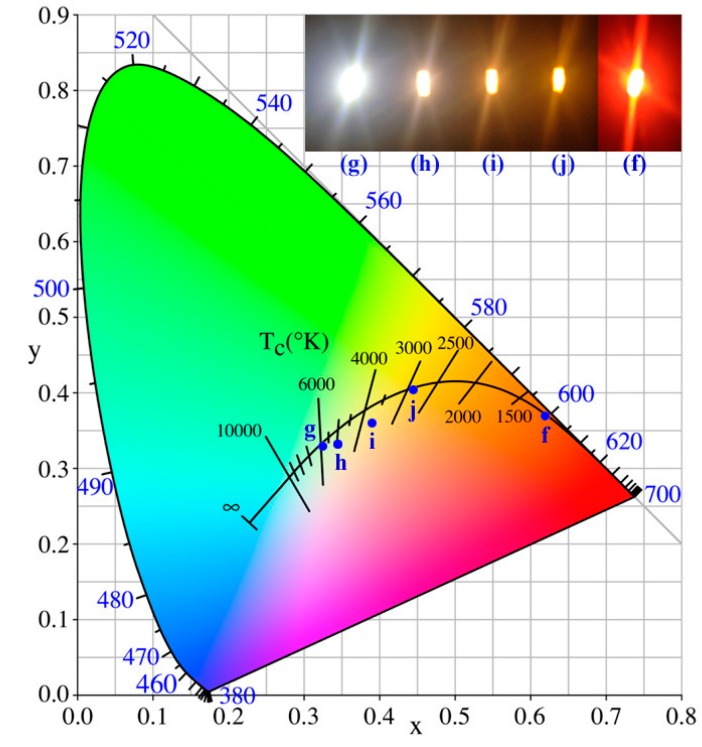
CIE chromaticity coordinates of blue GaN-based LEDs using YAG:Ce and [(TPTA)_2_Ir(dPPOA)]PF_6_ as phosphors at different blending concentrations. (**f**) only [(TPTA)_2_Ir(dPPOA)]PF_6_ at 6.0 wt %; (**g**) only YAG:Ce at 1.0 wt %; (**h**) 1.0 wt % YAG:Ce and 0.5 wt % [(TPTA)_2_Ir(dPPOA)]PF_6_; (**i**) 1.0 wt % YAG:Ce and 1.0 wt % [(TPTA)_2_Ir(dPPOA)]PF_6_; (**j**) 1.0 wt % YAG:Ce and 1.5 wt % [(TPTA)_2_Ir(dPPOA)]PF_6_. Inset: The photographs of the LEDs No. g, h, i, j and f in working state.

The luminous efficiencies of WLEDs No. h, i and j were85.2 lm∙W^−1^, 46.1 lm∙W^−1^ and 45.3 lm∙W^−1^ respectively. In contrast with WLED No. g (128.5 lm∙W^−1^), the luminous efficiencies decreased due to energy loss in light conversion. Besides some loss of luminous efficiencies when blue light was directly absorbed by [(TPTA)_2_Ir(dPPOA)]PF_6_ and transformed into orange light, some of the yellow light emitted from YAG:Ce was also absorbed by [(TPTA)_2_Ir(dPPOA)]PF_6_. Consequently, there was also a loss of luminous. Due to some degree of overlap between the excitation spectra of the iridium complex in silicone and the emission spectra of YAG:Ce (Figure 2 and Figure 3), such secondary light energy conversion aggravated the loss of total luminous efficiencies of the LEDs. By comparison with [Ir(CBT)_2_(dPPOA)]PF_6_ which was also used as a red-light-source phosphor in warm WLEDs in our previous work [15], the WLEDs using [Ir(CBT)_2_(dPPOA)]PF_6_ exhibited lower loss of luminous efficiencies. Thus, the aforementioned secondary light energy conversion between YAG:Ce and the iridium(III) complex can be ignored, mainly because ligand TPTA make wider excitation spectra for [(TPTA)_2_Ir(dPPOA)]PF_6_ than that of CBT for [Ir(CBT)_2_(dPPOA)]PF_6_. The excitation spectra of [Ir(CBT)_2_(dPPOA)]PF_6_ have much lower overlap with the emission spectra of YAG:Ce. Even so, the remaining efficiencies were still encouraging. Moreover, the CRIs of No. h, i and j have been improved, the CCTs of No. h, i and j have been lowered significantly and warmer white lights are obtained. The light emitted from WLED No. h became neutral white light, while the light emitted from WLEDs No. i and j were both warm white light. The CIE chromaticity coordinates of WLEDs No. g, h, i and j were (0.32, 0.33), (0.35, 0.33), (0.39, 0.36) and (0.44, 0.40), respectively. Their CIE chromaticity coordinates and working state photographs are all shown in Figure 9 to visually exhibit the aforementioned changes. In order to further exhibit the changes, the CIE chromaticity coordinates and working state photograph of the orange LED (No. f, only using [(TPTA)_2_Ir(dPPOA)]PF_6_ as a phosphor at 6.0 wt %) is also shown in Figure 9, because WLEDs No. h, i and j can be regarded as intermediate devices between WLEDs No. h and orange LED No. f.

## 4. Conclusions

A novel orange cationic iridium(III) complex [(TPTA)_2_Ir(dPPOA)]PF_6_ was synthesized. The [(TPTA)_2_Ir(dPPOA)]PF_6_ complex has a high thermal stability with a decomposition temperature of 375 °C, and its relative emission intensity at 100 °C is 88.8% of that at 25 °C. The [(TPTA)_2_Ir(dPPOA)]PF_6_ complex can be efficiently excited by blue GaN (450 nm) chips. GaN-based cold white LEDs using only YAG:Ce as a phosphor can become neutral WLEDs and warm WLEDs when [(TPTA)_2_Ir(dPPOA)]PF_6_ is added at proper concentrations. The [(TPTA)_2_Ir(dPPOA)]PF_6_ complex can effectively improve the red light component for WLEDs, and is a promising phosphor candidate for warm WLEDs.

## Figures and Tables

**Table 1:** Performances of blue GaN-based LEDs only using [(TPTA)_2_Ir(dPPOA)]PF_6_ as a phosphor at different blending concentrations (at 20 mA forward current).

No. of LEDs	Blending Concentration (wt %)	Luminous Efficiency (lm∙W^−1^)	CRI	CCT (K)	λ_em, max_ (nm)	CIE (*x*, *y*)
a	1.0	18.9	31.6	100,000	451, 609	(0.31, 0.17)
b	2.0	15.8	40.7	2159	451, 610	(0.37, 0.21)
c	3.0	12.1	46.6	1957	451, 614	(0.41, 0.25)
d	4.0	10.1	50.2	1737	451, 617	(0.45, 0.28)
e	5.0	7.3	51.6	1507	451, 619	(0.55, 0.35)
f	6.0	5.4	48.0	1265	619	(0.62, 0.38)

**Table 2:** Performances of WLEDs fabricated by using YAG:Ce and [(TPTA)_2_Ir(dPPOA)]PF_6_ as phosphors (at 20 mA forward current).

No. of LEDs	Blending Concentration (wt %)		Luminous Efficiency (lm∙W^−1^)	CRI	CCT (K)	λ_em, max_ (nm)	CIE (*x*, *y*)
	YAG:Ce	Complex					
g	1.0	0.0	128.5	72.2	6877	449, 556	(0.32, 0.33)
h	1.0	0.5	85.2	78.4	4896	448, 573	(0.35, 0.33)
i	1.0	1.0	46.1	80.2	3473	451, 583	(0.39, 0.36)
j	1.0	1.5	45.3	75.7	2864	450, 586	(0.44, 0.40)

## Supplementary Materials

The following are available online at www.mdpi.com/1996-1944/10/6/657/s1, Figure S1: ^1^H NMR spectrum of [(TPTA)_2_Ir(POA)]PF_6_, Figure S2: MS spectrum of [(TPTA)_2_Ir(POA)]PF_6_.

## References

[1] Pimputkar S., Speck J.S., DenBaars S.P., Nakamura S. (2009). Prospects for LED lighting. Nat. Photonics.

[2] Tsao J.Y., Crawford M.H., Coltrin M.E., Fischer A.J., Koleske D.D., Subramania G.S., Wang G.T., Wierer J.J., Karlicek R.F. (2014). Toward smart and ultra-efficient solid-state lighting. Adv. Opt. Mater..

[3] Feezell D.F., Speck J.S., DenBaars S.P., Nakamura S. (2013). Semipolar (20-2-1) InGaN/GaN light-emitting diodes for high-efficiency solid-state lighting. J. Disp. Technol..

[4] Chen L., Lin C.C., Yeh C.W., Liu R.S. (2010). Light converting inorganic phosphors for white light-emitting diodes. Materials.

[5] Jang H.S., Won Y.-H., Jeon D.Y. (2009). Improvement of electroluminescent property of blue LED coated with highly luminescent yellow-emitting phosphors. Appl. Phys. B.

[6] Zhu H., Lin C.C., Luo W., Shu S., Liu Z., Liu Y., Kong J., Ma E., Cao Y., Liu R.-S. (2014). Highly efficient non-rare-earth red emitting phosphor for warm white light-emitting diodes. Nat. Commun..

[7] Kwak H.H., Kim S.J., Park Y.S., Yoon H.H., Park S.J., Choi H.W. (2009). Photoluminescence Characteristic of Ce^3+^-Eu^3+^ Co-doped Y_3_Al_5_O_12_ Phosphor Prepared by Combustion Method. Mol. Cryst. Liq. Cryst..

[8] Jang H.S., Im W.B., Lee D.C., Jeon D.Y., Kim S.S. (2007). Enhancement of red spectral emission intensity of Y_3_Al_5_O_12_:Ce^3+^ phosphor via Pr Co-doping and Tb substitution for the application to white LEDs. J. Lumin..

[9] Wang X., Zhou G., Zhang H.L., Li H., Zhang Z., Sun Z. (2012). Luminescent properties of yellowish orange Y_3_Al_5−x_Si_x_O_12−x_N_x_: Ce phosphors and their applications in warm white light-emitting diodes. J. Alloys Compd..

[10] Lin C.C., Zheng Y.S., Chen H.Y., Ruan C.H., Xiao G.W., Liu R.S. (2010). Improving optical properties of white LED fabricated by a blue LED chip with yellow/red phosphors. J. Electrochem. Soc..

[11] Kim S., Kim T., Kang M., Kwak S.K., Yoo T.W., Park L.S., Yang I., Hwang S., Lee J.E., Kim S.K. (2012). Highly luminescent InP/GaP/ZnS nanocrystals and their application to white light-emitting diodes. J. Am. Chem. Soc..

[12] Zhou Q., Tan H., Zhou Y., Zhang Q., Wang Z., Yan J., Wu M. (2016). Optical performance of Mn^4+^ in a new hexa-coordinated fluorozirconate complex of Cs_2_ZrF_6_. J. Mater. Chem. C.

[13] Tan H., Rong M., Zhou Y., Yang Z., Wang Z., Zhang Q., Wang Q., Zhou Q. (2016). Luminescence behaviour of Mn^4+^ ions in seven coordination environments of K_3_ZrF_7_. Dalton Trans..

[14] Wang H., He P., Liu S., Shi J., Gong M. (2009). A europium(III) organic ternary complex applied in fabrication of near UV-based white light-emitting diodes. Appl. Phys. B.

[15] Meng G., Chen Z., Tang H., Liu Y., Wei L., Wang Z. (2015). A novel cationic iridium(III) complex as red phosphor applied in warm white light-emitting diodes. New J. Chem..

[16] Luo Y., Yan Q., Zhang Z., Yu X., Wu W., Su W., Zhang Q. (2009). White LED based on poly(N-vinylcarbazole) and lanthanide complexes ternary co-doping system. J. Photochem. Photobiol. A.

[17] Xiang H.-F., Yu S.-C., Che C.-M., Lai P.T. (2003). Efficient white and red light emission from GaN/tris-(8-hydroxyquinolato) aluminum/platinum(II) meso-tetrakis(pentafluorophenyl) porphyrin hybrid light-emitting diodes. Appl. Phys. Lett..

[18] Niklaus L., Dakhil H., Kostrzewa M., Coto P.B., Sonnewald U., Wierschem A., Costa R.D. (2016). Easy and versatile coating approach for long-living white hybrid light-emitting diodes. Mater. Horiz..

[19] Zhang C., Heeger A.J. (1998). Gallium nitride/conjugated polymer hybrid light emitting diodes: Performance and lifetime. J. Appl. Phys..

[20] Martino D.D., Beverina L., Sassi M., Brovelli S., Tubino R., Meinardi F. (2014). Straightforward fabrication of stable white LEDs by embedding of inorganic UV-LEDs into bulk polymerized polymethyl-methacrylate doped with organic dyes. Sci. Rep..

[21] Jin J.-Y., Kim H.-G., Hong C.-H., Suh E.-K., Lee Y.-S. (2007). White light emission from a blue LED, combined with a sodium salt of fluorescein dye. Synth. Met..

[22] Costa R.D., Ortί E., Bolink H.J., Monti F., Accorsi G., Armaroli N. (2012). Luminescent ionic transition-metal complexes for light-emitting electrochemical cells. Angew. Chem. Int. Ed..

[23] Hu T., He L., Duan L., Qiu Y. (2012). Solid-state light-emitting electrochemical cells based on ionic iridium(III) comp lexes. J. Mater. Chem..

[24] Ma D., Tsuboi T., Qiu Y., Duan L. (2017). Recent progress in ionic iridium(III) complexes for organic electronic devices. Adv. Mater..

[25] Tang H., Li Y., Chen Q., Chen B., Qiao Q., Yang W., Wu H., Cao Y. (2014). Efficient yellow-green light-emitting cationic iridium complexes based on 1,10-phenanthroline derivatives containing oxadiazol-triphenylamine unit. Dyes Pigments.

[26] Tang H., Li Y., Zhao B., Yang W., Wu H., Cao Y. (2012). Two novel orange cationic iridium(III) complexes with multifunctional ancillary ligands used for polymer light-emitting diodes. Org. Electron..

[27] Lo K.K.-W., Li S.P.-Y., Zhang K.Y. (2011). Development of luminescent iridium(III) polypyridine complexes as chemical and biological probes. New J. Chem..

[28] Yang Y., Zhao Q., Feng W., Li F. (2013). Luminescent chemodosimeters for bioimaging. Chem. Rev..

[29] Sun C.-Y., Wang X.-L., Zhang X., Qin C., Li P., Su Z.-M., Zhu D.-X., Shan G.-G., Shao K.-Z., Wu H. (2013). Efficient and tunable white-light emission of metal-organic frameworks by iridium-complex encapsulation. Nat. Commun..

[30] Yang H., Meng G., Zhou Y., Tang H., Zhao J., Wang Z. (2015). The photoluminescent properties of new cationic iridium (III) complexes using different anions and their applications in white light-emitting diodes. Materials.

[31] Dong H., Zhu H., Meng Q., Gong X., Hu W. (2012). Organic photoresponse materials and devices. Chem. Soc. Rev..

[32] Lin Y., Li Y., Zhan X. (2012). Small molecule semiconductors for high-efficiency organic photovoltaics. Chem. Soc. Rev..

[33] Chen X., Liu R., Xu Y., Zou G. (2012). Tunable protic ionic liquids as solvent-catalysts for improved synthesis of multiply substituted 1,2,4-triazoles from oxadiazoles and organoamines. Tetrahedron.

[34] Lowry M.S., Hudson W.R., Pascal R.A., Bernhard S. (2004). Accelerated luminophore discovery through combinatorial synthesis. J. Am. Chem. Soc..

[35] Lamansky S., Djurovich P., Murphy D., Abdel-Razzaq F., Lee H.-E., Adachi C., Burrows P.E., Forrest S.R., Thompson M.E. (2001). Highly phosphorescent bis-cyclometalated iridium complexes: Synthesis, photophysical characterization, and use in organic light emitting diodes. J. Am. Chem. Soc..

[36] Wong W.-Y., Zhou G.-J., Yu X.-M., Kwok H.-S., Lin Z.-Y. (2007). Efficient organic light-emitting diodes based on sublimable charged iridium phosphorescent emitters. Adv. Funct. Mater..

[37] Sahoo H. (2011). Föster resonance energy transfer—A spectroscopic nanoruler: Principle and applications. J. Photochem. Photobiol. C.

[38] Lü W., Lv W., Zhao Q., Jiao M., Shao B., You H. (2014). A novel efficient Mn^4+^ activated Ca_14_Al_10_Zn_6_O_35_ phosphor: Application in red-emitting and white LEDs. Inorg. Chem..

[39] Lee S.-P., Chan T.-S., Chen T.-M. (2015). Novel reddish-orange-emitting BaLa_2_Si_2_S_8_:Eu^2+^ thiosilicate phosphor for LED lighting. ACS Appl. Mater. Interfaces.

[40] Ruan J., Xie R.-J., Hirosaki N., Takeda T. (2011). Nitrogen gas pressure synthesis and photoluminescent properties of orange-red SrAlSi_4_N_7_:Eu^2^^+^ phosphors for white light-emitting diodes. J. Am. Ceram. Soc..

[41] Li H., Zhao R., Jia Y., Sun W., Fu J., Jiang L., Zhang S., Pang R., Li C. (2014). Sr_1.7_Zn_0.3_CeO_4_:Eu^3+^ novel red-emitting phosphors: Synthesis and photoluminescence properties. ACS Appl. Mater. Interfaces.

[42] Setlur A.A., Lyons R.J., Murphy J.E., Kumar N.P., Kishore M.S. (2013). Blue light-emitting diode phosphors based upon oxide, oxyhalide, and halide hosts. ECS J. Solid State Sci. Technol..

