# Supplementary material for: Warm White Light-Emitting Diodes Based on a Novel Orange Cationic Iridium(III) Complex
Source: Materials (Basel). 2017 Jun 16;10(6):657. doi: 10.3390/ma10060657 (PMC5554038; doi:10.3390/ma10060657)

Figure S1. ^{1}H NMR spectrum of [$(TPTA)_2Ir(POA)$]PF_6 ($CDCl_3$，400MHz)

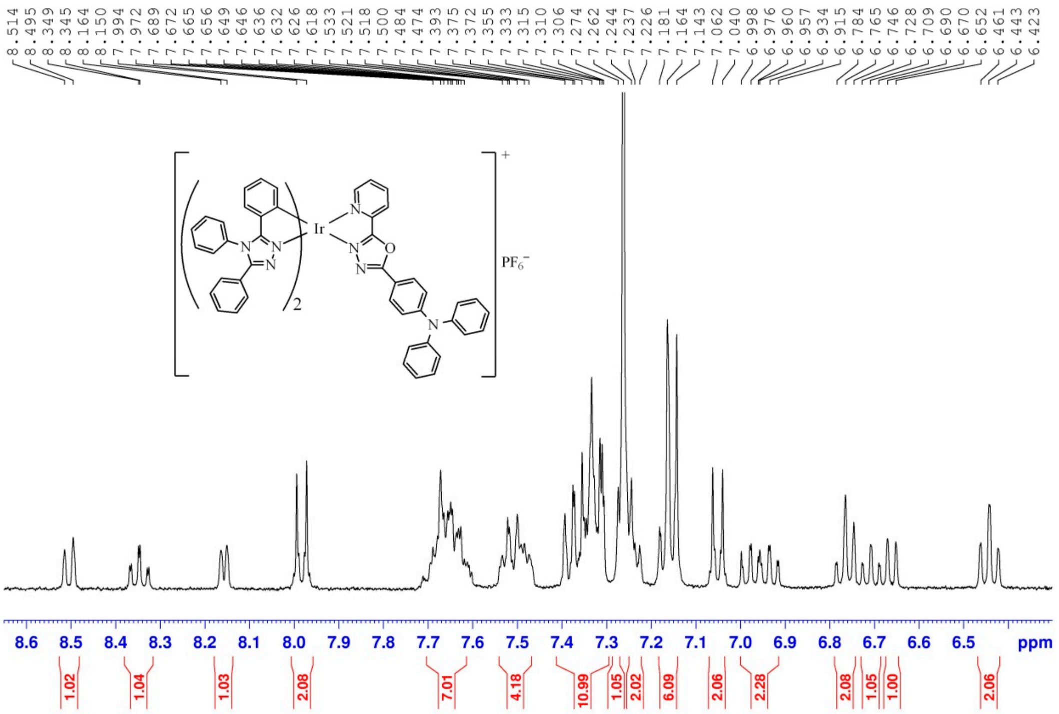

Figure S2. MS spectrum of [$(TPTA)_2Ir(POA)$]PF_6

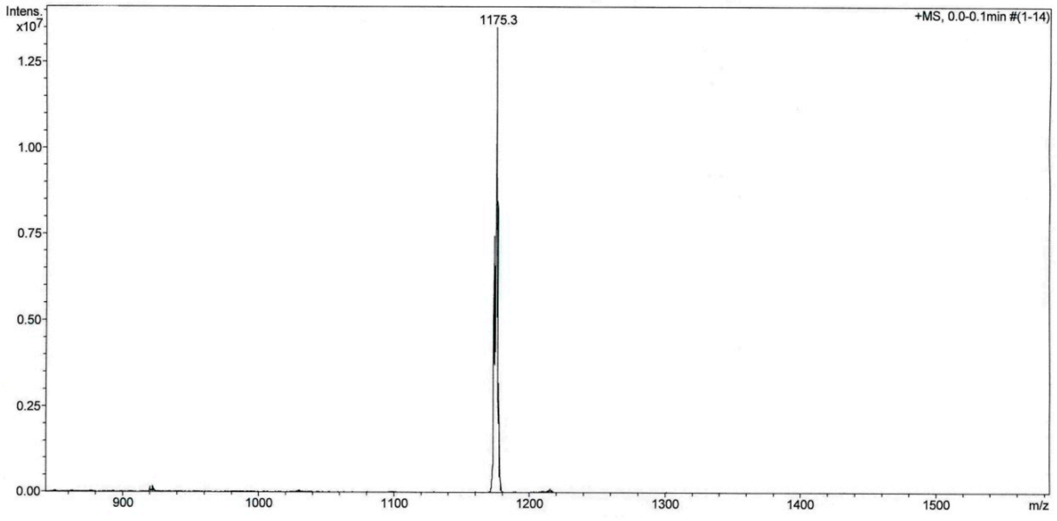

Supplement: Supplementary file 1 [file materials-10-00657-s001.pdf]
